# The role of kinship in bi-national couples: Intergenerational solidarity in Turkish-German families

**DOI:** 10.3389/fsoc.2022.856178

**Published:** 2022-09-01

**Authors:** Rena Tecklenburg, Mandy Boehnke

**Affiliations:** Bremen International Graduate School of Social Sciences, University of Bremen, Bremen, Germany

**Keywords:** bi-national couples, kinship, intergenerational solidarity, Turkish-German families, qualitative research, interethnic couples

## Abstract

Intermarriages, i.e., marriages between people from different ethnic backgrounds, have increased in recent years in many parts of the world and also in Germany. These marriages, often between an immigrant and a native partner, have various implications for family dynamics. To date, research has focused on the causes of ethnic exogamy, partnership quality, and fertility among interethnic couples. Using problem-centered interviews with Turkish-German couples living in Germany, the present study aims to broaden this perspective by looking at kin relationships (modes of interaction, spatial and emotional closeness, assistance and support, agreement on values and responsibilities), which have hardly been studied in bi-national families so far. The study pursues a qualitative research design that allows tracing kinship relations and perceived negotiation processes. Using the intergenerational solidarity typology as an heuristic for the qualitative content analysis the results will provide answers to the question what role kinship networks play in these partnerships and how their possibly different demands are balanced. The analysis of the interviews shows that in most cases the quality of relationships is high in both Turkish and German kinship networks and is characterized by openness and cordiality. As a result, relatives from both sides offer emotional, financial, or childcare support to the interviewed families, but differ in the type of support mainly due to physical proximity. Based on the results, we cannot claim that family cohesion is generally closer on one side of the extended family.

## Introduction

There have already been numerous studies on the relationship between parents and children using the intergenerational solidarity paradigm (e.g., Szydlik, 2008; Steinbach, 2008; Silverstein et al., 2010) and also migrant families are increasingly considered in the last decade (e.g., Bordone and de Valk, 2016; Albertini et al., 2019), while bi-national or interethnic couples have hardly ever been included so far. Against the background that bi-national couples are gaining in numerical importance, this is something to be changed. Intermarriages, i.e., marriages between people from different ethnic backgrounds, have increased in recent years in many parts of the world and also in Germany (Baykara-Krumme, 2020). These marriages, often between an immigrant and a native partner, have various implications for family dynamics. This study refers to couples in which one partner has a Turkish and the other a German background. This combination is of particular interest given that the largest group of so called guest workers Germany recruited between 1955 and 1973 came from Turkey. Even today, this is the most numerous combination of immigrants and native partners in intermarriages in Germany: Among bi-national marriages in Germany, marriages with Turkish partners are the frontrunner; in 2019, of the 381,514 marriages in Germany, 7,600 had Turkish husbands and 5,060 had wives with Turkish citizenship (Federal Statistical Office, 2022). We start with the assumption that Turkish-German couples and their families of origin show different family-related attitudes and expectations. This could lead to more conflicts both within the couple but also among the kin, which in turn could lead to less cohesion with the family. Previous studies have shown that marriage or cohabitation with a partner who has a different cultural and religious background can be more conflictual (Hohmann-Marriott and Amato, 2008) and has a higher risk of divorce (Milewski and Kulu, 2014). In addition, intermarriages also experience less support from relatives and family members (Kalmijn et al., 2005), which may be due to proximity but also lack of acceptance or sanctions (Kalmijn, 1998). We are interested in the specific conditions of bi-national families (married couples with children) and their interactions with the respective family networks. This is illustrated by qualitative data on different types of connectedness and solidarity (e.g., in the form of joint activities, agreement on values, support in care, emotional closeness).

On terminology: There are different terms for marriages composed of immigrants and natives; they have been called intermarriages, mixed marriages, interethnic marriages, or bi-national marriages. The term “bi-national marriage” narrows the concept to marriages between partners of different national origins, while “interethnic marriage” refers to marriages in which the spouses belong to different ethnic, religious, or national groups. This paper focuses on the relationship between Turkish-German couples and their parents as well as other relatives, we will use bi-national couples or interethnic couples interchangeably^1^.

The contribution is structured as follows. First, theoretical considerations and previous research in the field are presented, then the data collection is described. Results of the data analysis along solidarity dimensions form the core of the paper, which will conclude with a summary discussion.

## Theoretical considerations and previous research

This study draws on scholarship in two areas. The first is literature on intergenerational solidarity, the second research on immigrant families and intermarriage. The starting point for the first research stream is the model of intergenerational solidarity (Bengtson and Roberts, 1991) which distinguishes six dimensions of solidarity. These include aspects like shared activities (*associational* solidarity), emotional closeness (*affectual* solidarity), agreement on values (*consensual* solidarity), exchange of assistance (*functional* solidarity), filial obligations (*normative* solidarity) and geographical proximity (*structural* solidarity) (see Table 1 for an overview of the dimensions).

**Table 1:** Six elements of intergenerational solidarity with nominal definitions.

**Construct**	**Nominal definition**
Structural solidarity	Opportunity structure of intergenerational relationships reflected in number, type, and geographical proximity of family members
Associational solidarity	Frequency and patterns of interactions in various types of activities in which family members engage
Affectual solidarity	Type and degree of positive sentiments held about family members, and the degree of reciprocity of these sentiments
Consensual solidarity	Degree of agreement of values, attitudes, and beliefs among family members
Normative solidarity	Strength of commitment of performance of familial roles and to meeting family obligations (familism)
Functional solidarity	Degree of helping and exchanges of resources

Source: Adapted from Bengtson and Roberts (1991, p. 857).

Since the typology exists, it has invited many studies, looking at all (e.g., Lowenstein, 2007) or only selected dimensions (e.g., Tomassini et al., 2004), focusing on specific countries (e.g., Van Gaalen and Dykstra, 2006) or working comparatively (e.g., Lowenstein, 2007), looking at migrants (e.g., Albertini et al., 2019) and comparing them with non-migrants (of the same country of origin) (e.g., Baykara-Krumme and Fokkema, 2019) or natives in the country of residence (e.g., Rooyackers et al., 2014). It is this latter type of research that we will relate to as it is somewhat connected to our focus. Also of interest are those studies that target specific types of family relationships, such as those formulated by Silverstein and Bengtson (1997). Based on the dimensions of intergenerational solidarity they find five types that are typical for relations with both, mothers and fathers: tight-knit, sociable, intimate but distant, obligatory, and detached. Later, and based on the discussion of ambivalence (e.g., Lüscher, 2002), typologies have been developed that take into account both the positive and negative elements of intergenerational relationships. Conflict as an inherent aspect of family relationships was included. Overall, the model has proven to be a useful conceptual tool that is also appropriate for understanding family relationships in different countries, although it was developed for the study of grandparent-grandchild relationships in the United States. Because it has a rich history of study, we will also use it, but more in the sense of a heuristic. Conflict is not considered as an additional dimension, but as something that is assessed in conjunction with the other dimensions, taking into account what Bengtson et al. (2002) argue, namely that conflict is not the same as the absence of, for example, affection. The implicit consideration of conflict will hopefully prove helpful in understanding the dynamic processes of solidarity, ambivalence, and conflict in parent-child relationships.

Various theoretical and empirical research has shown differences between Western and non-Western family relationships and the different meaning of kinship in these societies based on different contexts (Kaǧıtçıbaşı, 1996). Markus and Kitayama (1991), who coined the concept of individualism-collectivism in social psychology, have argued that the Western self is constructed as independent from others, whereas people from non-western cultures construct interdependent selves, based on the fundamental relatedness of individuals to their in-group. Therefore, in Western countries, prevailing cultural norms promote individual autonomy, whereas in contrast, non-Western societies tend to be based on relatedness and family has a central position. Based on the assumption that norms are acquired through early socialization, Turkish immigrants are expected to rather be raised with close family ties, whereas Germans are more often raised to become independent. In bi-national relationships, this can lead to conflicts due to differences in how families are treated and what families expect from the couple and their offspring. In addition, because we have interviewed Turkish-German couples in Germany, the proximity of Turkish kin and the influence of migration must be taken into account. According to the solidarity paradigm opportunity structure of intergenerational relationships (structural solidarity) influences frequency and patterns of interactions (associational solidarity). Previous research on transnational family relationships raised two points in this regard: On the one hand, transnational families found to be having less close intergenerational cohesion than families living spatially closer together (Baykara-Krumme and Fokkema, 2019). On the other hand, spatial proximity does not seem to play a major role in the exchange of support and solidarity because of today's communication technologies (Baldassar, 2007).

In addition to cultural contexts individual characteristics influence parent-child relationships and possibly relationships to other extended kin. Dykstra and Fokkema (2011) examine variations in intergenerational late-life family solidarity in different countries. Their typology shows that mainly socio-demographic differences determine the type of support. Support from-a-distance (frequent contact but not living nearby) is more common in families with high incomes. With higher educational attainment of the adult children, the probability for the autonomous type (not living nearby, little contact, few support) and less ascending support increases. Additionally, the age of couples' children and couples' parents or other extended kin also might have an impact on intergenerational solidarity. With advanced age of parents and the need for upward support, e.g., health related functional support might increase. Previous studies showed that at the individual level, care of parents depends essentially on the needs of the parents, e.g., state of health (Haberkern and Szydlik, 2008). Downward functional support on the other hand, especially in form of care work, might be increased in families with young children (Hank and Buber, 2009). Last but not least, gender is also frequently mentioned as important indicator: Older parents are much more likely to be cared for by their daughters than by their sons (Leopold et al., 2014), and grandmothers are more active in grandchildren's care (Craig and Jenkins, 2016). In the next chapter we will present our study design before we continue with a description of the results sorted by the six dimensions of solidarity as described above.

## Materials and methods

### Study design

This paper is derived from research conducted as part of a larger project on value change and value transmission. Problem-centered interviews (Witzel and Reiter, 2012) with Turkish-German families are used to trace the role kinship networks play in interethnic partnerships. This method is a semi-structured, guideline supported narrative interview. The interviews were conducted with the two spouses and their children (if they were older than eight). All family members were interviewed together to observe and capture communication and negotiation processes within the couple and between generations. Family interviews allow to investigate how family members talk and reflect on kin relations and relationship quality. Thereby communication and negotiation processes between family members can be observed. Data collection took place between March and July 2020 and was challenged by the COVID-19 pandemic and its consequences.

### Sampling

In order to draw the appropriate sample of Turkish-German couples for the study, population registration offices were contacted who hold information on all residents of a given area. Among other things, their nationality and place of birth are recorded and linked to spouses (see also Glowsky, 2013). Several population registration offices in large cities were contacted, only Hamburg agreed to provide its data to meet the needs of this study. Using an onomastic technique a suitable sample was chosen from a provided list of 7,237 addresses of spouses with partners born in Turkey, respectively, Germany. This condition was not only important to have a potential high range of values between the partners but also increased the possibility of transnational relations to their respective families.

The aim was to study families in which both parents grew up in different socialization contexts and spent their formative years in Germany and Turkey, respectively. Eventually, 567 families were invited to participate in the qualitative interviews, of which 65 families responded to the initial invitation. Some of these 65 families had to be excluded because they did not meet all the required criteria. Selection criteria were that the Turkish partners spent their formative years in Turkey and the German partners in Germany, and that the couples have children. A selection was made from the 43 families who met the required criteria and agreed to participate in an interview. One goal was to ensure that the gender distribution of Turkish parents was balanced. The fact that more Turkish men are married to German women than vice versa was also evident in the families that were open to being interviewed. Therefore, the few families with a Turkish wife were selected first. A total of 17 interviews were conducted^2^. The participants (in case of children legal guardians) provided written informed consent to participate in this study.

### Interview guide

The interview guide contained various themes and sections that arose from the project context. The research guiding themes are essentially organized around three questions: first, how did both partners themselves grow up, second what is important to them in life, and third, how do they pass that on to their children? Themes of the interview guide were family and kinship, transmission, transitions, gender role attitudes, cultural aspects, everyday life, demographics, migration history, and living conditions. In this article, we only focus on the relevant sections of family relations and kinship. The interview guide and its specific narrative prompts were pretested with several interethnic parents.

After the interview, a postscript was written to record the atmosphere of the interview, special incidents, and the interviewer's impression. All interviews were conducted in German. For one interview, it was necessary to translate for a father from Turkish into German. This was done by his wife and daughter during the interview itself.

### Participants

As shown in Table 2, the sample consists of 17 families, each with one to four children aged one to 27. Four families have grown-up children, in seven families children are adolescent, and in six families, children are preschooler age or toddlers. Children of nine families also took part in the interview—if they were above eight and available (six daughters, four sons). In one family, only the mother and daughter were interviewed, this interview was not included in the analysis. The age of the spouses ranges from 34 to 68 years. In most cases, wives were slightly younger than their husbands. In twelve families, the husband held Turkish citizenship, and in five families, the wife did.

**Table 2:** Overview of participating families.

**No**.	**Age range wife**	**Education wife**	**Age range husband**	**Education husband**	**Children (sex and age range)**
1	50–55	Higher commercial school	50–55**	University degree	Daughter, 20–25
2	35–40*	University degree	40–45	PhD degree	Daughter, 5–10
3	50–55	University degree	45–50*	High school degree	Son, 15–20
4	65–70	University degree	55–60*	High School degree	Daughter, 20–25
5	55–60	High school degree, vocational training	50–55*	Middle school degree, vocational training	Daughter, 10–15
6	30–35	University degree	35–40*	University degree	Son, 0–5
7	40–45*	Middle school degree, vocational training	40–45	Middle school degree, vocational training	3 sons and a daughter, 5–15
8	35–40*	University degree	40–45	University degree	Son, 0–5
9	50–55	University degree	60–65*	University degree	2 daughters, 10–15
10	50–55	High school diploma, vocational training	50–55**	Middle school degree, vocational training	Son, 10–15
11	50–55	High school diploma, vocational training	50–55*	High school diploma	Son, 15–20
12	35–40*	University degree	35–40	University Degree	Son, 0–5
13	40–45	High school diploma, vocational training	40–45*	High school diploma, vocational training	2 sons, 0–5
14	45–50*	University degree	45–50	University degree	Son, 10–15
15	35–40	University degree	50–55*	Middle school, vocational training	Daughter, 5–10
16	40–45	High school diploma	45–50**	Middle school diploma, vocational training	Son and daughter, 5–15
17	55–60	High school diploma, vocational training	60–65*	University degree	2 sons, 20–30

^*^Turkish citizenship.

^**^Turkish citizenship; grew up mainly in Turkey but lived some years in Germany.

Although a condition for participation in this study was that both parents spent their formative years in Germany and Turkey, respectively, in three families the Turkish partner had moved back and forth between Turkey and Germany during childhood because their parents had lived in Germany as guest workers (sometimes referred to as the “Kofferkind” phenomenon). Families were still included if the Turkish partner spent the majority of their childhood and adolescence in Turkey. Parents of two Turkish partners still live in Germany. The parents of one German spouse live in another European country. In three families the parents of the German spouse live in the same house or even same flat as the interviewed family. Overall, a relatively high level of education and socioeconomic status can be observed in the families. Half of the spouses hold a university degree (see Table 2 for an overview of participating families). Ten families live in rented accommodation, seven families own their homes.

### Data analysis

The analysis followed a deductive-inductive approach. The interviews were transcribed and analyzed in a data-driven manner based on qualitative content analysis (Schreier, 2012; Kuckartz, 2014) supported by MaxQDA software. During the transcription and accompanying the analysis process, memos were written that contained contextual information and initial interpretations (Witzel and Reiter, 2012). Following the steps of qualitative content analysis, a “basic coding and reconstruction of prior interpretations” (Witzel and Reiter, 2012, p. 102f) took place and a code system was created. The interviewer mainly coded all interviews with support of a coding group consisting of coders who were part of the research project but did not collect data and interviews. This sharing of categories increases intercoder reliability. The coding system was applied to each interview and supplemented with new categories and subcategories as new interviews were integrated. All texts were analyzed and interpreted vertically. This was followed by the horizontal analysis and the main interpretation phase. Two main categories, namely “contact with family of origin” and “role of extended family”, were first assigned by both authors to Bengtson and Roberts' (1991) six solidarity dimensions. Thereafter, the assigned codes were extracted and summarized. Descriptions of the similarities and differences within the families are the main component of the result presentation. The goal is to present the specifics of solidarity in interethnic families. Different from most other research on family solidarity, in this research not only older parents and their adult children but also their (grand-) children and other family members are to some extent part of the analysis. However, the analysis is based on the perspective of the interethnic couple. We will therefore not address reciprocity of the described relationship, which is part of original model. The distribution of the sample by gender, age and origin will be considered in analyzing the organization of intergenerational support.

The period during which the study was conducted was challenging for intergenerational relations due to the COVID-19 pandemic. Some contact between households was limited due to quarantine measures. This circumstance had a slight impact on habitual interactions between family members living either in Germany or abroad. For example, flights were on hold and visits to Turkey not possible for a few months. Also, the data collection had to be interrupted for two months.

## Results

The following part presenting our empirical results is structured by the six solidarity types as defined by Bengtson and Roberts (1991) and described above.

### Structural solidarity

Structural solidarity, which sets the opportunities for family members to interact with each other, is embedded in special circumstances for bi-national families. In this study the relatives of the Turkish side do not live in Germany in most cases, whereas the relatives of the German side do.

As earlier mentioned in the description of the sample, some of the Turkish parents live or used to live in Germany, of which one parental couple continues to live in the same country as their child. In two other families Turkish parents are divorced and only one parent stayed in Germany. However, most of the Turkish parents live in Turkey. Nine German parents live nearby, in the same city or even in the same house as their children. On the contrary, eight German children live away from their parents, sometimes far, e.g., in rural southern Germany. This particular setting, in which the relatives live in different countries, forms the basis and particularity of the family studied here.

As we will see in the next sections, regular contact with parents and other extended family members was reported more frequently with those living in Germany than in Turkey. In some families, this means that close contact was not established between parents and their grandchildren. In one interview, it was mentioned that the community aspect was fundamentally stronger in Turkey. Not only parents and other relatives would contribute as support in caring for young children, but also neighbors and acquaintances from the family network.

In a few cases, there is evidence that a parent's health condition or financial situation plays a role in how often support is provided and what kind of intergenerational solidarity is possible. It also has an impact on overall contact between parents and children. Visits involve higher costs for families whose kin live in Turkey, as they usually take more time and flights have to be paid for. This presents a financial challenge for some families. One way to mitigate this challenge is to combine family vacations with family visits. This is discussed in more detail in the section on associational solidarity below.

The COVID-19 pandemic changed the frequency of contact between generations slightly, although the impact of contact restrictions were not addressed often within the interviews. Keeping in touch with parents in Turkey *via* videophone over different messengers, which were already established, enabled to maintain the frequency and mode of interaction. Flights to Turkey to visit parents on the other hand had to be postponed until further notice. A lack of social network was particularly evident during this time, however, often in terms of distance to both families in Turkey and Germany.

### Associational solidarity

Most of the interviewees reported contact with their parents, but also with other relatives, such as siblings, nieces and nephews, aunts and uncles.

The families interviewed typically visit the Turkish part of their family of origin regularly during vacations, usually once a year. Personal contacts with parents living nearby in Germany take place more frequently than with family members in Turkey. This includes contacts between grandchildren and their grandparents, e.g., joint vacations or overnight stays. Frequent contact also takes place in the form of telephone calls, online messages or video calls. These contacts are more frequent with the Turkish relatives, up to weekly contacts.

It is striking that statements about interactions with the German family were made less frequently compared to the Turkish side of the family. As already described, some of the Turkish relatives live in Germany, which has an influence on the frequency and occurs more regularly. Some families do not have close contact with their Turkish parents and have not visited each other for several years. In a few families, visits do occur, but more out of obligation to the older generation. Holidays are typically celebrated with all generations together. At least parents of both origins are called at these special occasions. This was mentioned for Christmas or Eid al-Fitr (Ramazan Bayrami) and Eid al-Adha (Kurban Bayrami). In two families, it was reported that the Turkish partner in the bi-national family visits his or her parents in Turkey even without the spouse and children and outside of a vacation. In many families, parents or siblings of the Turkish partner come to visit, sometimes regularly every year, sometimes less frequently on special occasions. Contact with the German side of the family is more regular, which is also due to the fact that these families live closer together (sometimes in the same house). However, spatial distance is not the only determining factor, as the travel distance from Turkey is sometimes at least in theory shorter than from Germany, for example, if the parents live in rural regions in southern Germany. The following interview summarizes the complexity.

“Well, we live here far off the beaten path. Both in terms of my family and his family. Theoretically, the distance between my husband's parents and Hamburg is closer because the flight time is only 3.5–4 hours and to my sisters and to my mother it is 5.5–6 hours by train. For both of us family is very important I would say. We often have difficulties here or start floundering, so not only that family is important to us, but also now with regard to our son we would find it nice if he could spend more time with aunts, uncles and cousin, who are in Turkey. But that is currently not possible. […] And we have nevertheless the luck that my sisters comes for example very often to visit. My brother even lives here, but has his own family and is very involved.” (Female partner of the interethnic couple, German, Interview 6: 3)

### Affectual solidarity

In most families, intergenerational relationships are characterized by warmth and affection, which is particularly emphasized describing the Turkish side. Close ties are also maintained with siblings. Despite initial reservations about the interethnic relationship of their children, in most cases the sons or daughters-in-law were warmly welcomed into the extended families. In rare cases, the bond between kin and interviewed families is less strong. Although there are annual visits between them, the relationship is less close and warm than in most others. The relationship between the respective in-laws is rarely mentioned. When it is mentioned, the reinforcing effect on cohesion within the entire family is emphasized.

Close family relationships are described with both extended families, those in Turkey as well as those located in Germany. But associating family relations with warmth occurs less frequently in German families than in Turkish ones. While emotional closeness is emphasized in the context of Turkish families, granting freedom to pursue own goals is mentioned more frequently in German families. The different expression of affection can be traced in the following quotations.

“From the Turkish side, I feel that there is a close family bond. Also physically, that you hug each other. And the grandparents are always open for that as well.” (Female partner of the interethnic couple, German, Interview 17: 80)

“My parents gave me a lot of freedom to make decisions within certain limits. I also moved [away], for example, and later, I married in Turkey. And my parents would have been happy if I had stayed nearby, but they also gave me the freedom. And I visited them as often as possible and talked to them on the phone and wrote to them and sent photos. And we still have a good relationship.” (Female partner of the interethnic couple, German, Interview 5: 81)

### Consensual solidarity

It was striking that the interviewed couples more often described disagreements with their parents due to different beliefs and attitudes than they reported agreements. It became clear that these differences were much more important at the beginning of the Turkish-German relationship than in later years, often based on the parents' reservations about their offspring's interethnic marriage. In addition to the frequently mentioned uneasiness of the parents, due to prejudices against the nationality of the child-in-law, especially different religious affiliations met with reservations. This was more prevalent on the Turkish than on the German side of the family. The grown-up children therefore either compromised and feigned religious conversion to their parents or disobeyed their parents' instructions. Consensus between parents and children was reported in the way holidays are celebrated when rituals are adopted and holidays are celebrated together. In two cases, children performed a ritual wedding for the sake of their parents. To not upset their father (-in-law) his daughter and son-in-law only pretend to practice the Muslim religion.

In retrospect, most parents are satisfied with their offspring's interethnic marriage when they see them living in a happy relationship and getting to know their in-laws. Despite the differences in everyday practices, which are especially noticeable at visits, the acceptance of being different is high among both generations. Another topic where parents' and children's ideas do not always correspond are parenting issues, particularly when grandchildren are young. In some cases, parents of the interviewed families hold views on parenting that the spouses do not agree with. The circumcision of grandsons was a frequent cause for disagreement. In this case, too, the children usually flout their parents' ideas.

R1: “And the doctor looked at my son [for a possible circumcision], and then my son cried. Because he was little. But it wasn't because he was crying. Somehow I had the feeling that I was doing something bad to him, […] And then I had somehow, how do you say, protective instincts. It's not supposed to be that way. Then I turned against my family.

R2: When we were on vacation, [his parents] asked, ‘And when are you having the circumcision party?' And my husband said that's not possible with him. The doctor said it won't work for him.

R1: It's like that. Like I said, if the doctor doesn't approve it, then it's not [done]. […] Actually, I don't think it was okay for my parents, but they couldn't change it. I said this is my child, this is my decision.”

(R1: Male partner of the interethnic couple, Turkish, R2: Female partner, German, Interview 3: 240–242)

### Normative solidarity

In addition to family values, we also subsumed gender role attitudes, expectations and norms regarding marriage and religion, and parent-child-roles into this solidarity category.

Families show gender role attitudes across the spectrum: Traditional role models are promoted by the interviewed spouses as well as egalitarian ones. Country of origin seems to play a subordinate role within the couples. However, traditional expectations are more frequently demanded by Turkish relatives than by German ones. Demands for the appropriate partner regarding marrying a Muslim were expressed outright from Turkish fathers toward their daughters in two cases.

“My dad is deeply religious, he was. And he loved him [my husband] very much and liked him and so. But becoming Muslim, in our culture, maybe you already know that. A woman can't marry with a Christian or a Jew and so, if he didn't become Muslim before. You can't. So before that you have to (convert). But if a Muslim man wants to marry a Christian or Jew or something, he can convert later. But a woman is not allowed to (marry) before. And that was a motto for my dad. So one should and so. Then we did, played along.” (Female partner of the interethnic couple, Turkish, Interview 14: 22)

To this day, the couple pretends to the Turkish family that the German husband has converted to Islam. They feign conversion to Islam in order to conform to their father's religious ideas. Other couple handles these gaps in attitudes differently. For example, they adapt their behavior in their parents' environment. Normative expectations also exist from the German side, but they are mentioned less frequently and seem to be less strong. In addition, adjustments are made less frequently when the parents are present.

In one family, both partners adapt to the gender expectations of their respective in-laws: The German daughter-in-law subordinates herself to the Turkish family and the Turkish son-in-law entertains guests at German family celebrations.

“In the meantime, we've become such a well-coordinated team. You can tell. If I'm in the vicinity of the Turkish family, or if they're guests here, then I just do what the Turkish woman does. Everything. Or in Turkey, I subordinate myself to them. But when we meet the German side or are invited, it is often the case that my husband stands up and asks who would like a beer? And fetches the drinks. And he does that immediately. That we know exactly what each side actually wants.” (Female partner of the interethnic couple, German, Interview 16: 430–433)

### Functional solidarity

Overall, functional solidarity is mentioned more frequently for the German side of the kinship than for the Turkish side. Functional solidarity can be differentiated in the type and direction of support, for the later distinguishing between upward support (i.e., help from the child to the parent) and downward support (help from the parent to the child) might be helpful (Bordone and de Valk, 2016).

In Turkish families, it is rather the parents who support their children, so-called downward support. For German parents, upward support was more often indicated. As Table 2 shows, six families have children of toddler age, seven families have teenaged children and in five families children are grown up. This can also be used to estimate the care needs of the (in-law) parents of the couples. In two families, German mothers need daily care because of health issues. They are living together with their child's family. Overall, due to physical proximity to German kin, there is more often daily support, like in childcare for grandchildren or household chores. Support from the Turkish side of the family is less prominent, but occurs on special occasions. Maternal support was reported after births, financing and organizing the circumcision feast as well as advice in upbringing methods. The type of functional support, upward as well as downward, reveals classical gender division of labor. In all cases, the wife takes over the care for German parents in case of illness, no matter if she cares for her own parents. It is also sisters or mothers who come regularly or for visits to assist with child care. Sons (in-law) and fathers (in-law) rather take over the organization of celebrations and practical help.

Denied support was also mentioned when Turkish parents tried to impose their will. One example of denied downward support was a daughter's demand that both grandparents should not interfere in her grandson's religious education. In a single family, mutual support either with Turkish or German kin does not take place, even if it would be spatially possible, since Turkish and German relatives live nearby. This is due to personal preferences.

Living far away from their own parents is more often a source of worry for the Turkish partner in the couple. Especially when they get older and their need for help increases. The following quote shows that it is difficult to maintain support between the generations in a transnational setting. The son would like to support his parents, which is often not possible due to the distance. To change this circumstance, a move to Turkey is discussed between the couple and considered as an option for retirement.

“They're not the youngest either, in one situation or another I would have said, being there at that moment would have been good, that you can just settle that, because so to speak I'm the oldest son, and the first grandchild of 6, 7 children, and that I then have to slowly take over my father's role. And that also comes for my wife, she also has to slowly take over my mother's role. My father is the oldest, and he must take care of many things. And then sometimes my father wishes that I am there, and in some situations I also wish that I am there.” (Male partner of the interethnic couple, Turkish, Interview 3: 327–329)

## Discussion and conclusion

Our aim is to contribute to the existing literature by considering solidarity types in a transnational space in bi-national families. The results illustrate that there are indeed differences between Turkish and German extended family members in terms of intergenerational support. Conflicts between generations due to the interethnic relationship of the partners do occur. However, these were mainly observed in consensual and partly normative solidarity. Conflicts arose within families mainly due to parental reservations at the beginning of interethnic relations. The unknown fueled skepticism. Especially daughters are confronted with these challenges. The families interviewed seem to have found their way to deal with such challenges and the solution seems to lie in compromise.

As previous studies have shown, cross-border distance from part of the family makes a difference, namely in functional solidarity. Here we found more day-to-day support from and for the German side of the kin. This becomes especially evident when parents get older and need more assistance. Also, the gendered division of care work is evident in the sample, independent from the origin of spouses. In terms of affectual solidarity, however, we did not find much difference, although physical affection was more emphasized with the Turkish side of the kin. Associational solidarity is mainly lived in different forms, which can be attributed to the different distance to the parents. One could say that with Turkish family members, contact over distance is replaced with new technologies. Instead of being able to see each other physically on a regular basis, families use video calls and messages to keep in touch which is in line with research by Baldassar (2007).

As far as family cohesion is concerned, it cannot be stated that it is generally closer on one side of the extended family. In individual cases, the connection with the Turkish parents is not as close as with the German parents. Language was mentioned by the interviewees as a possible reason for the close relationship between the generations. Being able to speak the same language can create more closeness in the relationship between grandparents and grandchildren, but was not always a prerequisite for a close relationship. Cohesion in German families tends to be expressed through concrete support. Regardless of the country of origin, we found close and less close relationships in the sample.

Selection effects of the interviewed families certainly play a role for several of the observed dimensions of solidarity. The couples decided to live in Germany rather than in Turkey, which is often explained by the social and political structures in Turkey and job opportunities in Germany. Thus, they are less likely to be confronted with everyday role expectations from the Turkish side, but also receive less daily support. For future research, it would be informative to compare the families interviewed in this study with interethnic couples who chose to live in Turkey. In contrast to Bengtson and Roberts (1991) and other studies that focus on associational solidarity in their research, the structural dimension seems to play a central role in bi-national couples due to the transnational nature of kinship relations.

## Data availability statement

The raw data supporting the conclusions of this article will be made available by the authors, without undue reservation.

## Ethics statement

The studies involving human participants were reviewed and approved by Ethics Committee Jacobs University Bremen. Written informed consent to participate in this study was provided by the participants' legal guardian/next of kin. Written informed consent was obtained from the individuals and the minors' legal guardians for the publication of the anonymised data contained in this article.

## Author contributions

MB conceptualized the paper, wrote sections of the text, and provided interpretation of the results. RT was responsible for data collection, did the main part of the analysis, and wrote sections of the text. Both authors contributed to the article and approved the submitted version.

## Funding

We thank University of Bremen for their generous funding of the project.

## Conflict of interest

The authors declare that the research was conducted in the absence of any commercial or financial relationships that could be construed as a potential conflict of interest.

## Publisher's note

All claims expressed in this article are solely those of the authors and do not necessarily represent those of their affiliated organizations, or those of the publisher, the editors and the reviewers. Any product that may be evaluated in this article, or claim that may be made by its manufacturer, is not guaranteed or endorsed by the publisher.

## Footnotes

^1^We are aware that the German part of Turkish-German couples in Germany are often second generation migrants with German citizenship, in this paper we are interested in interethnic couples and selected couples accordingly. We will pick up this point when describing our sample selection.

^2^The “point of diminishing return” (Mason, 2010 adapted from Breuer et al., 2019, p. 159) was reached and no new insights are expected to emerge when additional families are interviewed.
